# The Potential Hypoglycemic Competence of Low Molecular Weight Polysaccharides Obtained from *Laminaria japonica*

**DOI:** 10.3390/foods12203809

**Published:** 2023-10-17

**Authors:** Aijun Tong, Dengwei Wang, Xiaoyan Liu, Zhiqun Li, Runfan Zhao, Bin Liu, Chao Zhao

**Affiliations:** 1 College of Food Science, Fujian Agriculture and Forestry University, Fuzhou 350002, China; tajlove@163.com (A.T.); lizhiqun0595@163.com (Z.L.); 2 Marine Food Research and Development Center, Fuzhou Ocean Research Institute, Fuzhou 350002, China; 3 Department of Chronic and Noncommunicable Disease Control and Prevention, Fujian Provincial Center for Disease Control and Prevention, Fuzhou 350012, China; wdw2023@126.com; 4 Beijing Engineering and Technology Research Center of Food Additives, School of Food and Health, Beijing Technology and Business University, Beijing 100048, China; liuxiaoyan8112@163.com; 5 College of Life Science, Fujian Agriculture and Forestry University, Fuzhou 350002, China; xiaozhufan20230907@163.com; 6 College of Marine Sciences, Fujian Agriculture and Forestry University, Fuzhou 350002, China

**Keywords:** low molecular weight, *Laminaria japonica* polysaccharide, type 2 diabetes, gut microbiota

## Abstract

This study aimed to assess the hypoglycemic efficacy of low molecular weight polysaccharides fractions obtained from *Laminaria japonica* (LJOO) in a model of type 2 diabetes mellitus (T2DM) constructed using mice. Biochemical parameters were measured after 4 weeks of continuous gavage, and fasting blood glucose (FBG) concentrations were analyzed. Pathological changes in tissues were assessed. The intestinal contents were obtained for 16S rDNA high-throughput sequencing analysis and detection of short-chain fatty acids (SCFAs). LJOO lowered FBG and insulin concentrations. It altered the gut microbiota composition, as evidenced by enriched probiotic bacteria, along with an increase in the Bacteroidetes/Firmicutes ratio and a decrease in the population of harmful bacteria. LJOO stimulated the growth of SCFA—producing bacteria, thereby increasing cecal SCFAs levels. LJOO can potentially aid in alleviating T2DM and related gut microbiota dysbiosis. LJOO may be used as a food supplement for patients with T2DM.

## 1. Introduction

Among its prevalent forms, type 2 diabetes mellitus (T2DM), poses a risk to human health globally. Its characteristics include insulin resistance and hyperglycemia, and T2DM is associated with complications due to dysfunctional insulin secretion and impaired beta cell function [1]. Numerous studies have indicated a strong link between modifications of the gut microbiota and T2DM’s incidence [2]. Typical observations suggest that the Firmicutes-to-Bacteroidetes ratio (F/B) correlates positively with blood glucose concentrations, and is strongly associated with liver damage and insulin resistance, among other symptoms. Studies have also demonstrated that *Roseburia*, *Bifidobacterium*, *Akkermansia*, *Bacteroides*, and *Faecalibacterium* are negatively correlated with T2DM, whereas *Ruminococcus* and *Fusobacterium* show a positive correlation [3]. Mechanisms by which intestinal flora affect T2DM include facilitating intestinal appetite and inflammatory responses, with short-chain fatty acids (SCFAs) as mediators [4]. SCFAs support the proper functioning of the intestines, safeguard the structural integrity of colonic epithelial cells, regulate inflammation, and control the processes of digestion and absorption.

Some scholars have recently proposed and demonstrated that phytochemicals effectively improve intestinal flora balance to regulate immune and metabolic-related diseases. Polysaccharides exert hypolipidemic and hypoglycemic effects and mitigate lipid metabolism disorder. A significant correlation between the molecular weight of polysaccharides and their biological activity has been demonstrated [5]. *Enteromorpha prolifera* oligosaccharide can modify the diversity and composition of mouse gut microbiota, regulate blood glucose metabolism, and protect against aging [6]. *Laminaria japonica* is a widely cultivated marine macroalgae species found along coastlines and is used as both a food source and for its medicinal properties [7]. *Laminaria japonica* polysaccharides (LJOO) can restore blood sugar balance and improve lipid profile by altering the composition of intestinal flora [8]. Immune-active LJOO has a molecular weight ranging from 6 to 8 kDa [9]. Low molecular weight polysaccharide fractions show notable hypoglycemic effectiveness in mice with high glucose-induced insulin resistance [10].

However, the impacts and underlying mechanisms of low molecular weight polysaccharide action sourced from *Laminaria japonica* in mouse model remain undetermined. We employed a mouse model of T2DM to evaluate the effects of LJOO. The mechanisms of action of LJOO were investigated through biochemical and 16S rRNA gene sequencing analyses. The purpose of this work is to provide updated evidence and insights into the regulation of T2DM utilizing LJOO to offer potential avenues for future investigations and the development of therapeutic strategies to prevent and alleviate T2DM.

## 2. Materials and Methods

### 2.1. Reagents

The *L. japonica* variety, “Huangguan No. 1” (Lianjiang, China) was used. Indicated enzyme-linked immune sorbent assay (ELISA) kits (Wuhan Chundu Biotechnology Co., Ltd., Wuhan, China) were used to determine the concentrations of fasting insulin (FINS), interleukin (IL)-6, tumor necrosis factor α (TNF-α), nuclear factor-κ-gene binding (NF-κB), IL-10, and glucagon-likepeptide-1 (GLP-1). Corresponding standard assay kits (Jiancheng, Nanjing, China) were used for determining glycosylated serum protein (GSP), triglyceride (TG), high/low-density lipoprotein-cholesterol (HDL-C and LDL-C, respectively), and total cholesterol (TC) concentrations.

### 2.2. Preparation and Analysis of LJOO

First, dried *L. japonica* powder was dissolved twice in ultrapure water in 1:40 (*w*/*v*) ratio for 0.5 h. Next, the volume of the combined supernatant was made up to 75–80% (*v*/*v*) by ethanol addition. The crude polysaccharide was collected and further treated with 2% neutral protease to remove proteins. Subsequently, the supernatant containing crude polysaccharides was dialyzed for 48 h using an 8–14 kDa dialysis bag, and the dialyzed solution was treated with sulfuric acid (18.4 mol/L) and neutralized with sodium hydroxide (1 mol/L). The supernatant was subjected to concentration. After two days of dialysis using 1- and 5-kDa-cutoff membranes, the deproteinized supernatant was lyophilized and LJOO was consequently obtained. The powders of LJOO were individually combined with KBr in a ratio of 1:100 (*w*/*w*) and compressed to form a 1 mm thin layer, which was subjected to Fourier Transform Infrared Spectroscopy analysis (FT-IR, NEXUS-670, Nicolet, Madison, WI, USA). The monosaccharide composition of LJOO was evaluated using Ion Chromatography (ICS5000, Thermo Fisher, Waltham, MA, USA). A MALLS system together with a refractive index detector (Wyatt Technology, Santa Barbara, CA, USA) and gel permeation chromatography (GPC) (Agilent, Santa Clara, CA, USA) were used to determine the molecular weight of LJOO.

### 2.3. Animals and the Design of Experiments

ICR mice (male; 18–22 g) were kept in a clean, hygienic environment, with unrestricted access to water and normal feed. The mice were procured from Wu’s Laboratory Animal Co., Ltd., Fuzhou, China. The Academic Committee of Fujian Agriculture and Forestry University (PZCASFAFU21026) approved all experimental protocols. After adaptive feeding for one week, for construction of the T2DM model through the injection of streptozocin, 32 mice were used, and eight animals were chosen randomly as the normal control (NC) group and fed a standard chow diet [11]. The remaining mice were divided into three groups, with eight animals each as follows: Model, MET (treatment of diabetic mice with 100 mg/kg metformin), and LJOO treatment (treatment of diabetic mice either with 100 or 200 mg/kg LJOO, as indicated). Gavage was administered for 28 days. Water was provided daily to the animals in the NC and Model groups.

### 2.4. Sample Collection and Biochemical Assays

Fasting blood glucose (FBG) was determined using tail vein-collected blood after 12 h of fasting at weeks 0, 2, and 4 of gavage administration, as indicated. An oral glucose tolerance test (OGTT) was conducted by first measuring FBG, followed by 2 g/kg gavage of glucose solution, determining the blood glucose values after 30, 60, and 120 min of glucose gavage, and finally calculating the area under curve (AUC) for glucose tolerance. The animals were euthanized by cervical dislocation following blood collection from eyeballs. The cecal contents of the mice were collected and stored at −80 °C to subsequently analyze SCFAs and composition of intestinal microflora. Homeostasis model assessment insulin resistance index (HOMA-IRI) was estimated following the standard protocol. The formula for calculating HOMA-IRI using the serum insulin values is as follows: HOMA-IRI: FIN (mU/L) × FBG (mmol/L)/22.5. Standard assay kits were used to detect several biological indicators including GSP, GLP-1, IL-6, and TNF-α in the sera. Similarly, LDL-C, TC, HDL-C, and TG levels in the liver were determined.

### 2.5. Histopathological Analysis

Liver, cecal, and pancreatic tissues were fixed in 4% paraformaldehyde. Subsequently, fixed tissues were embedded in paraffin blocks and cut into 4 μm thick sections using a sectioning machine, followed by staining with hematoxylin-eosin (H&E) and treatment with xylene to obtain transparent sections. These sections were imaged under an optical microscope (Nikon, Tokyo, Japan).

### 2.6. Quantification of SCFAs

Cecal contents (100 mg) were diluted in 2 mL of distilled water to obtain a slurry, and 0.5 mL of H_3_PO_4_ and 1 mL of diethyl ether were added to it. The slurry was centrifuged at 4 °C and 7000 rpm for 10 min after vortexing for 4 min. A 0.22 µm microporous membrane was used to filter the organic phase. Gas chromatography (GC–MS; Agilent-8860, Santa Clara, CA, USA) was used to synthesize and determine concentrations of various SCFAs in organic fractions.

### 2.7. Sequencing of Cecal Microflora

The bacterial 16S rRNA was amplified using the universal primer pairs for the V3–V4 region, 806R and 341F, using the bacterial genomic DNA extracted from cecal contents. The Illumina TruSeq DNA PCR-Free Sample Preparation Kit was used to construct bacterial 16S rRNA gene sequencing libraries. Novogene Co., Ltd., Beijing, China performed high-throughput sequencing on the Illumina NovaSeq PE250 platform. The parameters were determined using the Qiime software (ver. 1.9.1); alpha diversity (Shannon, Simpson indexes) was estimated, and the “ggplot2” package was utilized to draw a cumulative box chart of species. LDA Effect Size (LEfSe) analysis was performed to estimate the relative abundances of gut microbiota between groups (*p* < 0.05, LDA > 3.0) and identify statistically significant microbiota genera for subsequent correlation analysis.

### 2.8. Statistical Analysis

One-way analysis of variance with Tukey’s correction was performed. Mean and standard deviation (SD) are presented for all experiments. *p* < 0.05 was defined as a statistically significant difference.

## 3. Results and Discussion

### 3.1. Characterization of LJOO Polysaccharides

**Figure 1:**
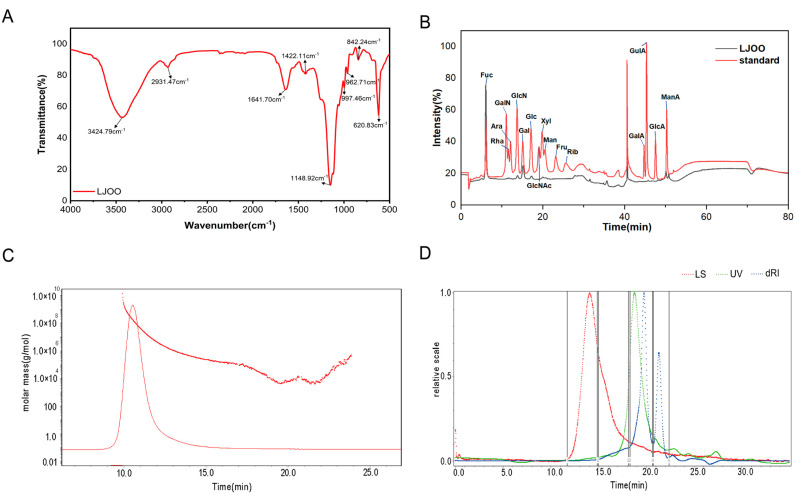
Decomposition of LJOO. (**A**) FT-IR spectra; (**B**) monosaccharide standard and composition; (**C**) absolute molecular weight analysis; (**D**) the homogeneity of LJOO. Abbreviations: fucoidan—Fuc, arabinose—Rha, N-Acetyl-D-Galactosamine hydrochloride—GalN, N-Acetyl-D-Glucosamine hydrochloride—GlcN, xylose—Ara, glucose—Glc, galactose—Gal, xylose—Xyl, N-Acetyl-D-Glucosamine—GlcNAc, fructose—Fru, mannose—Man, galacturonic acid—GalA, ribose-Rib, glucuronic acid—GulA, mannuronic acid—ManA, and glucuronic acid—GlcA. The parabolic curve indicates the multi-angle laser light scattering signal, where the scattering intensity is directly proportional to the molecular weight and size of the compound; the scattered line depicts the molecular weight obtained from fitting the two signals. dRI: differential refractive index detector.

The FT-IR spectra recorded at 4000–500 cm^−1^ were used to determine the structure of LJOO (Figure 1A). A strong absorption peak at 3424.79 cm^−1^ can be attributed to the O–H stretching of the sugar residues. The absorption peak at 2931.47 cm^−1^ (within the range of 3000–2800 cm^−1^) indicates the stretching of the methyl or methylene C–H bonds of the sugar residues. Similarly, the absorption peak at 1641.70 cm^−1^ indicates the stretching vibrations of deprotonated carboxyl groups (COO–) and C–H bonds. The absorption peak at 1148.92 cm^−1^ is indicative of the stretching of C–H bonds, a distinguishing fingerprint of oligosaccharides. Absorption peaks resulting from the stretching of S–O–S and C–O–S are at 620.83 cm^−1^ and 977.46 cm^−1^, respectively. Taken together, LJOO mainly comprises fucoidan, glucosamine hydrochloride, rhamnose, glucose, xylose, mannose, and mannuronic acid, in a molar ratio of 0.730:0.014:0.157:0.022:0.017:0.019:0.042. The functional activity of polysaccharides depends on their structural characteristics, such as the sulfation degree, types of monosaccharides, glycosidic branching, and distribution of molecular weights (Figure 1B) [5]. In this study, the molecular weights of LJOO fractions were determined using the GPC-RI-MALLS technique, and the average molecular weight (Mw) of LJOO was found to be 6.366 kDa (Figure 1C,D).

### 3.2. LJOO Polysaccharides’ Effects on the Glucose Metabolism of T2DM Mice

**Figure 2:**
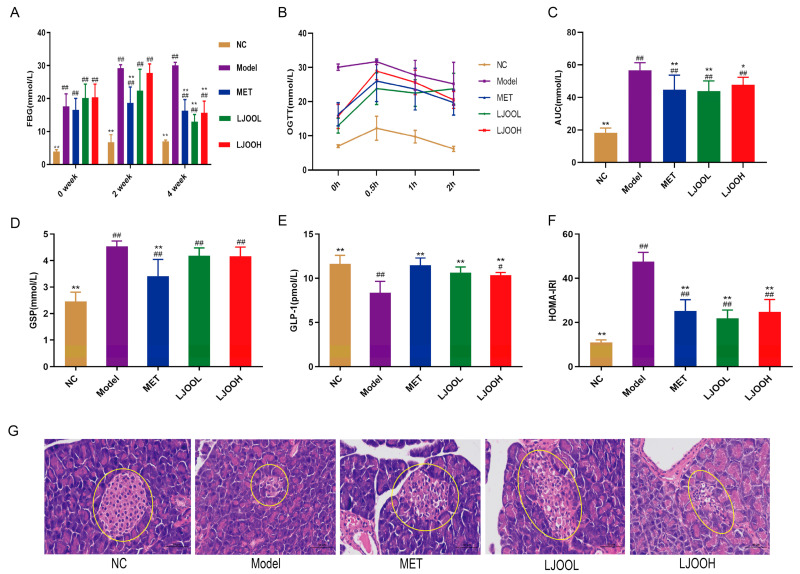
LJOO’s effects on glucose metabolism in T2DM mice. (**A**) FBG; (**B**) OGTT; (**C**) AUC; (**D**) GSP; (**E**) GLP-1; (**F**) HOMA-IRI; (**G**) pancreatic tissue (400× magnification). Note: ^#^ *p* < 0.05, ^##^ *p* < 0.01 vs. the NC group; * *p* < 0.05, ** *p* < 0.01 vs. the Model group.

The characteristics of T2DM include disrupted insulin and glucose metabolism, leading to insulin resistance and hyperglycemia, respectively [12]. Measurement of FBG is a strong and intuitive indicator for assessing the control of blood glucose concentrations by LJOO in diabetic mice. FBG levels in the diabetic group at week 0 were significantly higher than those in the NC group (*p* < 0.01), with concentrations exceeding 11.0 mmol/L, indicating a successful establishment of the T2DM model. Following two weeks of LJOO intervention, the MET and LJOO groups showed a decrease in FBG concentrations compared to the Model group, but did not reach statistical significance. However, by the fourth week, FBG concentration had significantly decreased in both groups except for the NC group, indicating a certain control effect of LJOO on blood glucose levels (Figure 2A). OGTT, which is performed to assess glucose tolerance, is a major diagnostic tool for diabetes. Blood glucose concentrations in all groups increased sharply after oral glucose administration, reaching a peak within 30 min, and gradually decreasing thereafter, returning to pre-glucose levels after 2 h. FBG levels in the Model group were significantly greater compared to those in the NC group at indicated time points, while the values in the other experimental groups lay in between those of the two groups (Figure 2B), indicating that the development of T2DM leads to impaired glucose tolerance in mice but there is a certain degree of recovery after intervention with MET and LJOO. The corresponding area under the curve (AUC) comprehensively details the changes in blood glucose levels. AUCs were compared with the corresponding values in the Model group, and both the MET and LJOO groups showed significantly lower values (*p* < 0.01) (Figure 2C). GSP effectively indicates the average blood glucose levels in mice in the last 1–2 weeks, unaffected by temporary fluctuations in blood glucose concentrations. GLP-1 has a glucose-dependent hypoglycemic effect [13]. The LJOO group showed reduced levels of GSP. Compared to the Model group, the LJOOL and LJOOH groups showed a significant increase in GLP-1 concentrations in the serum (*p* < 0.01) (Figure 2D,E). LJOO could induce high levels of GLP-1.

The HOMA homeostasis model utilizes the correlation between FBG and FINS levels as an indicator of the equilibrium between glucose metabolism and insulin secretion [14]. HOMA-IRI was employed for the assessment of insulin resistance [15]. Both LJOOL and LJOOH significantly reversed high HOMA-IRI levels due to a high-fat diet and streptozotocin (*p* < 0.01) (Figure 2F). H&E staining analysis of pancreatic tissue sections accurately and visually depicts the distribution of islet cells and the level of insulin secretion. The structure of pancreatic cells was found to be intact and clear in all groups, and the yellow-circled area in the NC group indicated the region of pancreatic islet cells, distributed in a cluster. The islet region in the Model group was significantly smaller with blurred borders unlike that in the NC group. Compared with the Model group, the islet areas in the MET and LJOO groups were significantly larger, and the whole islet structure was complete and better clustered (Figure 2G).

### 3.3. LJOO’s Effects on Liver Histopathology and Lipid Metabolism of T2DM Mice

**Figure 3:**
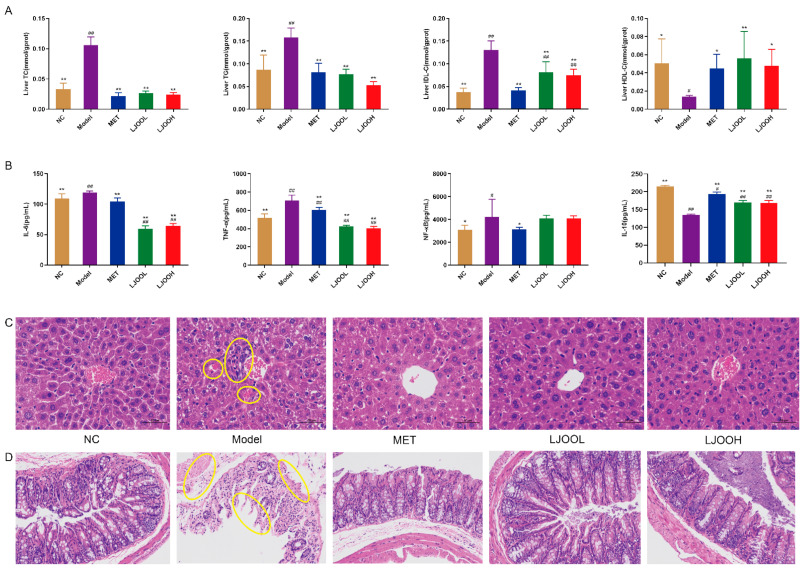
LJOO’s effect on lipid metabolism and anti-inflammatory molecules in T2DM mice. (**A**) Hepatic LDL-C, TG, HDL-C, and TC levels; (**B**) TNF-α, IL-6, IL-10, and NF-κB levels; (**C**) liver tissue (400× magnification); (**D**) cecal tissue (200× magnification). Note: ^#^ *p* < 0.05, ^##^ *p* < 0.01 vs. the NC group; * *p* < 0.05, ** *p* < 0.01 vs. the Model group.

Disturbed glucose metabolism in T2DM mice exacerbates liver tissue injury and results in altered liver function-related indices (LDL-C, TG, TC, and HDL-C) [16]. Compared with the NC group, hepatic LDL-C, TG, and TC concentrations were significantly higher (*p* < 0.01), while those of hepatic HDL-C were significantly lower (*p* < 0.05) in the Model group (Figure 3A). MET effectively lowered cholesterol, TC, TG, and LDL-C. The 200 mg/kg dose administered in the LJOOH group regulated TC, TG, and LDL-C levels more favorably, evidenced by significant alleviation by 77.87%, 66.23%, and 42.08%, respectively, after 4 weeks of treatment, in comparison with the Model group (*p* < 0.01). To further investigate the effect of LJOO, the fat in mouse livers was assessed by H&E staining (Figure 3C). At 400× magnification, hepatocyte gaps appeared larger, cells were loosened, and several cells were ruptured or underwent autolysis and necrosis; the number of lipid droplets in the liver was significantly greater in the Model group than that in the livers of mice in the NC group, while it was significantly lower in the LJOO group in comparison with the Model group, indicating that LJOO inhibited the accumulation of fat in the livers of mice. Taken together, the results indicate that LJOO can play a certain lipid-lowering role and prevent the formation of fatty liver.

### 3.4. LJOO’s Effects on Cecal Histopathology and Anti-Inflammatory Molecules in T2DM Mice

The elevation of specific inflammation markers, such as NF-κB, IL-6, TNF-α, and IL-10, plays an integral role in metabolic disorders. IL-6, TNF-α, and NF-κB can alter insulin sensitivity by stimulating crucial steps in the insulin signaling cascade [17]. Chronic inflammation is intimately linked to various diseases, including diabetes, atherosclerosis, and cancer. Natural polysaccharides exert anti-inflammatory effects [18]. After 4 weeks of treatment, serum IL-6 levels were significantly enhanced in the Model group than in the NC group (*p* < 0.01), while the reverse was observed for IL-10 concentration (*p* < 0.01). Serum TNF-α, NF-κB, and IL-6 levels were significantly lower in the MET, LJOOL, and LJOOH groups than in the Model group (*p* < 0.01), while significantly increased serum IL-10 expression (*p* < 0.01) was found compared to the Model group (Figure 3B). Taken together, LJOO exerted certain anti-inflammatory effects on T2DM mice. H&E staining of the cecum (Figure 3D) showed that the outer membrane of the cecal tissue, muscle mucosa, and villi were intact and clear in the NC group, and the inflammatory granulocyte infiltration in the intestinal mucosa and the destruction of the villi structure were more severe in the Model group than in the NC group. After 4 weeks of LJOO treatment, the number of villi in the LJOOH group increased significantly and were arranged in an orderly pattern. LJOO intervention conferred a protective effect against T2DM—induced intestinal inflammation. Huang et al. [19] reported that a polysaccharide extracted from the fruit body of *Sanghuangporus vaninii* could alleviate hyperglycemia and hyperlipidemia in mice with T2DM by exerting anti-inflammatory effects, and our findings are consistent.

### 3.5. LJOO’s Effects on SCFAs Concentrations in the Cecum

**Figure 4:**
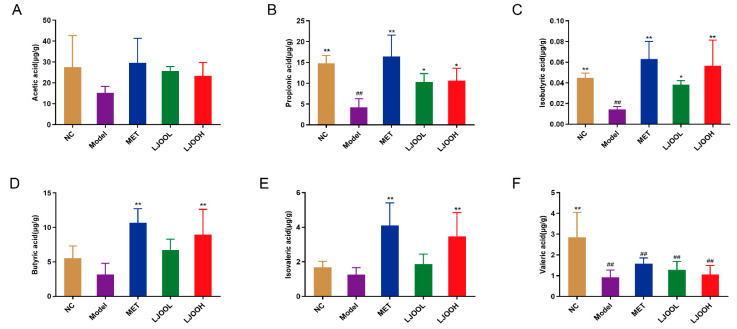
LJOO’s effects on cecal SCFAs concentrations. (**A**) Acetic acid; (**B**) propionic acid; (**C**) isobutyric acid; (**D**) butyric acid; (**E**) isovaleric acid; (**F**) valeric acid. Note: ^##^ *p* < 0.01 vs. the NC group; * *p* < 0.05, ** *p* < 0.01 vs. the Model group.

Gut bacterial metabolites such as SCFAs affect immune cells and the gut. They may serve as promising targets both in clinical treatment and the development of medications for diabetes [20]. SCFAs include acetic, isobutyric, butyric, propionic, valeric, and isovaleric acids. The contents of propionic, isobutyric, and valeric acid in the NC group were significantly enhanced than those in the Model group (*p* < 0.01) (Figure 4). After administrated of LJOOH for 4 weeks, the concentrations of propionic, isobutyric, butyric, and isovaleric acid concentrations were significantly higher than those in the Model group (*p* < 0.05), demonstrating that LJOO significantly upregulated SCFAs production by the gut microbiota.

### 3.6. LJOO’s Effects on Cecal Contents’ Compositions

**Figure 5:**
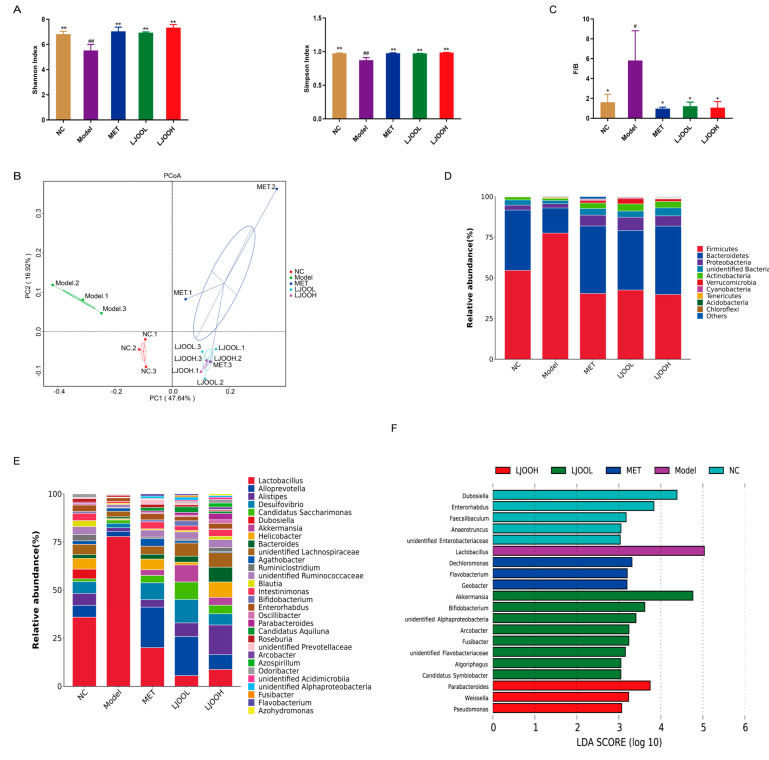
LJOO’s effects on the composition of cecal content. (**A**) Alpha diversity (Shannon and Simpson indexes); (**B**) beta diversity (PCoA); (**C**) F/B; (**D**) gut microbiota composition at the level of phylum; (**E**) gut microbiota composition at the level of the genus; (**F**) LEfSe analysis at the level of the genus (LDA > 3.0, *p* < 0.05). Note: ^#^ *p* < 0.05, ^##^ *p* < 0.01 vs. the NC group; * *p* < 0.05, ** *p* < 0.01 vs. the Model group.

The gut microbiota in humans is characterized by a degree of stability and diversity [21]. Abundant empirical and converging evidence supports the notion that gut microbiota plays a causal role in the regulation of glucose homeostasis [22]. Alpha diversity demonstrates microbial community diversity within a sample [23] (Figure 5A). Compared to the mice in the NC group, Shannon and Simpson indexes in the Model group reduced markedly while, in comparison with the Model group, LJOO administration caused a significant increase in gut microbiota diversity, as evidenced by higher values of the Shannon and Simpson indexes. Enhanced microbial richness and diversity were demonstrated by principal coordinate analysis (PCoA) for beta diversity, which compares the microbial community composition among samples [24]. The overall structure of the gut microbiota was analyzed by PCoA (Figure 5B), which demonstrated marked changes in the gut microbial composition following MET and LJOO treatment. The findings suggest that T2DM significantly alters the structure of the intestinal microflora, while LJOO potentially alleviates these effects.

The relative abundances of bacterial species were examined at both the phylum and genus levels to evaluate alterations in the microbial community. The phylum level was dominated by Bacteroidetes, Firmicutes, Proteobacteria, Verrucomicrobia, and Actinobacteria (Figure 5C). A primary cause that underlies metabolic disorders and insulin resistance is the rise in the F/B value. The F/B ratio is elevated in patients with T2DM [25]. The F/B values in the NC, MET, LJOOL, and LJOOH groups were 1.63, 0.98, 1.23, and 1.07, respectively, all significantly lower than the ratio in the Model group at 5.83 (*p* < 0.01) (Figure 5D). At the genus level, *Lactobacillus*, *Alloprevotella*, *Alistipes*, *Desulfovibri*, *Candidatus Saccharimonas*, *Dubosiella*, *Akkermansia*, *Helicobactera*, and *Bacteroides* were dominant. The abundances of *Lactobacillus* and *Agathobacter* increased while those of *Alloprevotella*, *Akkermansia*, *Ruminiclostridium*, *Dubosiella*, *Alistipes*, and *Bifidobacterium* decreased due to T2DM and, after LJOO treatment, the abundances showed some recovery (Figure 5E). The proportion of *Lactobacillus* in diabetic mice is known to be higher than that in the normal mice, consistent with the results of this study [26].

To further assess LJOO intervention’s effects on the representative microbiota in diabetic mice, LEfSe was employed to conduct genus-level linear discriminant analysis (LDA ≥ 3.0). Compared across different groups (NC, Model, MET, LJOOL, LJOOH), the results of LDA revealed 20 discriminant features at the level of the genus (Figure 5F). The five representative bacteria in the NC group were *Dubosiella*, *Enterorhabdus*, *Faecalibaculum*, *Anaerotruncus*, and *unidentified Enterobacteriaceae*. One representative bacteria of the Model group was *Lactobacillus*. Therefore, T2DM mice experienced alterations in gut dysbiosis and the composition of their intestinal microbiota. *Akkermansia*, *Bifidobacterium*, *Arcobacter*, *Fusibacter*, and *Algoriphagus* genera predominated among the gut microbiota of the LJOOL group, and concomitantly, *Parabacteroides*, *Weissella*, and *Pseudomonas* played a vital role in the LJOOH group. *Parabacteroides* and *Algoriphagus* belong to Bacteroidetes, and *Weissella* and *Fusibacter* belong to Firmicutes. Commonly reported findings indicate that *Akkermansia* and *Bifidobacterium* genera are negatively associated with T2DM [3]. *Parabacteroides* contribute to disease development including diabetes, obesity, inflammatory bowel syndrome, and autoimmune disorders [27]. *Weissella* has probiotics properties and produces several bioactive molecules including biogenic amines, bacteriocins, folate, and enzymes, among others [28]. Taken together, treatment with LJOO increased the abundance of dominant gut bacteria in mice, which may explain why LJOO helps lower blood sugar.

### 3.7. LJOO’s Effects on the Metabolic Functions of Cecal Contents

**Figure 6:**
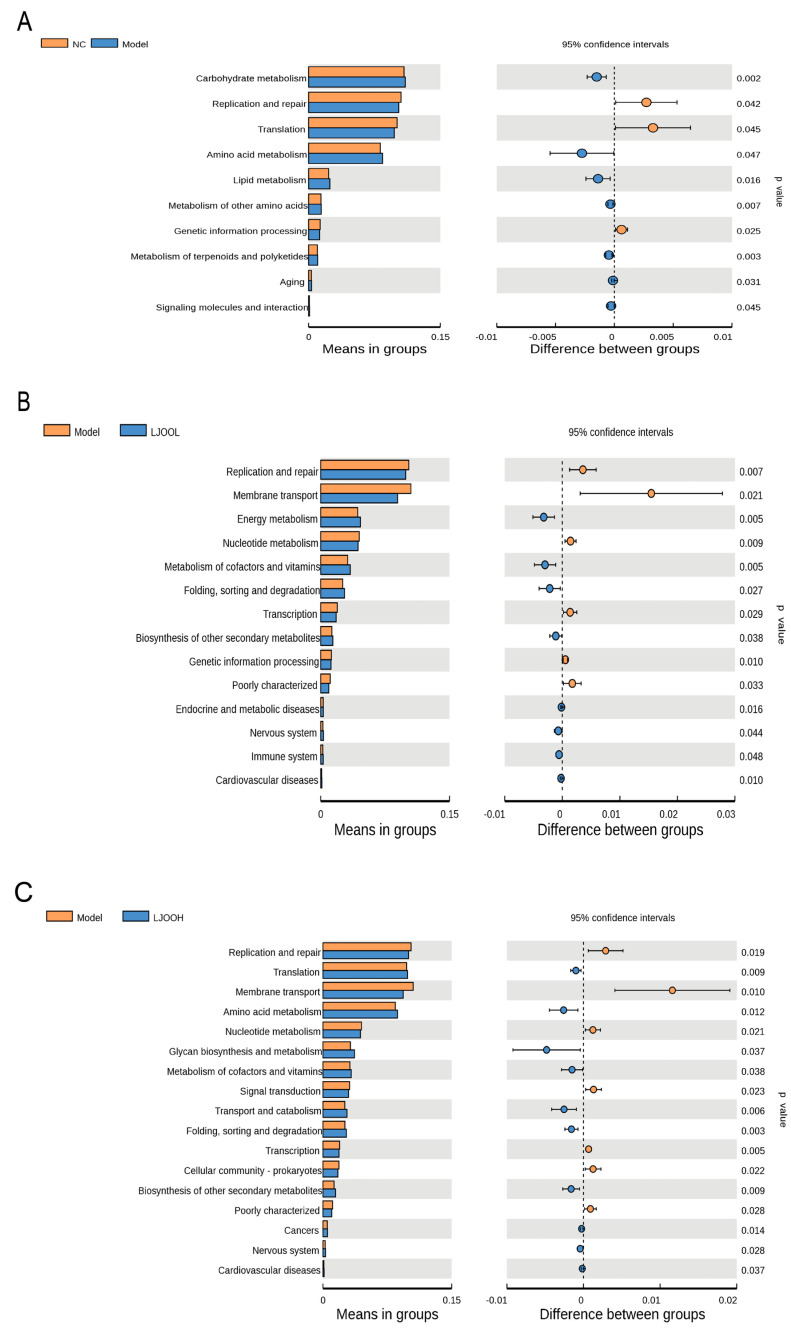
Functional prediction for the intestinal microbiota using Tax4Fun. (**A**) NC versus Model; (**B**) Model versus LJOOL; (**C**) Model versus LJOOH.

Using Tax4Fun, functional predictions for the microbial communities in each group were made. Tax4Fun is based on the 16S Silva database that is used for the functional prediction of gut samples with high accuracy [29]. By comparative analysis using the KEGG (level 2) database, metabolic functions of the gut microbiota across groups were analyzed. Given the annotated results from the database, a *t*-test differential analysis was simultaneously performed (Figure 6). Functional modules differed between NC and Model groups (*p* < 0.05), including carbohydrate metabolism, replication and repair, amino acid metabolism, and lipid metabolism. Fourteen significant (*p* < 0.05) and differential functional modules between the Model and LJOOL groups were identified, mainly including the biosynthesis of other secondary metabolites, endocrine and metabolic diseases, immune system, and cardiovascular diseases. The functional module related to cardiovascular diseases was significant and differential in the Model group compared with the LJOOH group (*p* < 0.05). Seventeen differentially functional modules were statistically significant (*p* < 0.05) between the Model and LJOOH groups. Cardiovascular diseases, including lipid and atherosclerosis and diabetic cardiomyopathy pathways, are closely associated with T2DM. These results hint potential mechanism of the beneficial effect of LJOO intervention through the regulation of endocrine, metabolic, and cardiovascular diseases induced by T2DM.

### 3.8. Correlational Analysis

**Figure 7:**
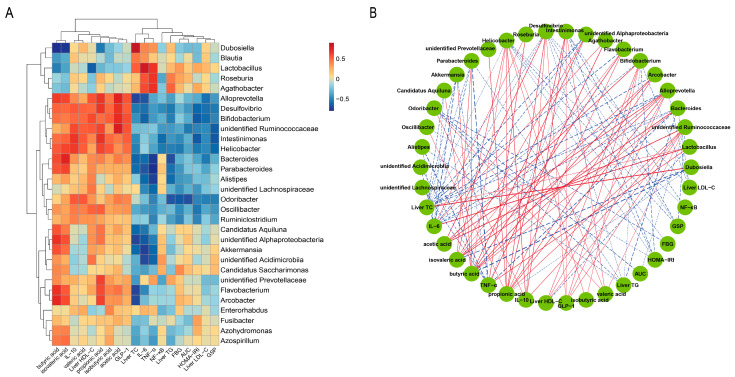
Spearman’s correlations for the genera present in the cecal microbiota of mice with other parameters. (**A**) Heatmap of hierarchical clustering shows the correlation between biochemical indicators, SCFAs and intestinal contents; (**B**) correlation network is shown, where solid red and dotted blue lines indicate positive and negative correlations, respectively (|r| > 0.5 and *p* < 0.05).

To better illustrate the hypoglycemic effect of LJOO, a Spearman correlation analysis was performed to examine the possible associations between gut bacterial abundance, host biochemical markers, and SCFAs concentrations (Figure 7A). The microbes belonging to *Bifidobacterium* and *Alloprevotella* were negatively correlated with FBG, AUC, GSP, HOMA-IRI, liver TC, liver TG, liver LDL-C, IL-6, NF-κB, and TNF-α levels, but were positively correlated with SCFAs levels. In contrast, the relative abundance of *Lactobacillus*, enriched in the Model group, depicted an opposite correlational trend with SCFAs and various biochemical markers. *Bifidobacterium* consists of anaerobic Gram-positive bacteria and constitutes a predominant genus within the gut microbiota that is important in promoting health-related properties [30]. *Bifidobacterium*, with probiotic properties, benefits host health, and some of its strains regulate SCFAs metabolism and the gut microbial composition [31]. Wei et al. [32] reported that intermittent administration of a tryptophan-deficient diet to T2DM mice can increase the relative abundance of *Alloprevotella* in the mouse intestines, thereby improving symptoms of hyperglycemia. LJOO could improve T2DM by modulating the relative abundances of *Bifidobacterium* and *Alloprevotella* and regulating SCFAs levels.

Correlational network and hierarchical clustering were visualized to examine the associations between biochemical markers, genera, and SCFAs (|r| > 0.5 and *p* < 0.05) (Figure 7B). *Dubosiella* belongs to the family Erysipelotrichidae and has one known species, *Dubosiella newyorkensis* found in the gut microbiota of mice [33]. There was an observed increase in *Dubosiella* after feeding on a high-fat diet [34]. It correlates positively with body weight gain and negatively with fecal SCFAs, suggesting its potential involvement in the progression of gut microbiota dysbiosis [35]. The LJOO (LJOOL and LJOOH) groups also showed fewer pathogenic *Dubosiella* with strong and positive correlation with liver TC, whereas a significant and negative association with the levels of butyric acid, isovaleric acid, and propionic acid. The elevated presence of *Dubosiella* could contribute to the development of hyperlipidemia and hyperglycemia. In previous studies, similar results were reported, wherein an increase in *Dubosiella* was observed in mice fed a high-fat diet [36].

## 4. Conclusions

We successfully isolated low molecular weight polysaccharide fractions from *Laminaria japonica* extracts. Results from animal experiments indicated that LJOO potentially benefits the improvement in the levels of FBG, AUC, HOMA-IRI, GLP-1, and inflammatory factors, along with hepatic lipid metabolism in mice induced by a high-sugar and high-fat diet. LJOO regulated intestinal flora composition, enriched the diversity of intestinal flora, and increased cecal SCFAs concentrations. Moreover, the results of 16S sequencing based on intestinal flora suggest that LJOO’s regulatory effect on blood sugar levels involves multiple metabolic pathways. However, the targets and mechanism of LJOO action in glucose and lipid metabolism need to be validated in further experiments.

## Data Availability

The data used to support the findings of this study can be made available by the corresponding author upon request.
